# Ziyuglycoside II Inhibits the Growth of Human Breast Carcinoma MDA-MB-435 Cells via Cell Cycle Arrest and Induction of Apoptosis through the Mitochondria Dependent Pathway

**DOI:** 10.3390/ijms140918041

**Published:** 2013-09-03

**Authors:** Xue Zhu, Ke Wang, Kai Zhang, Biao Huang, Jue Zhang, Yi Zhang, Lan Zhu, Bin Zhou, Fanfan Zhou

**Affiliations:** 1 Key Laboratory of Nuclear Medicine, Ministry of Health, Jiangsu Key Laboratory of Molecular Nuclear Medicine, Jiangsu Institute of Nuclear Medicine, Wuxi 214063, China; E-Mails: zhuxue0108@gmail.com (X.Z.); zhangkai@jsinm.org (K.Z.); huangbiao@jsinm.org (B.H.); zhangjue@jsinm.org (J.Z.); zhangyi@jsinm.org (Y.Z.); zhulan@jsinm.org (L.Z.); zhulan@jsinm.org (B.Z.); 2 Faculty of Pharmacy, The University of Sydney, Sydney, NSW 2006, Australia; E-Mail: fanfan.zhou@sydney.edu.au

**Keywords:** ziyuglycoside II, MDA-MB-435, cell cycle arrest, cell apoptosis

## Abstract

Ziyuglycoside II is one of the major active compounds of *Sanguisorba officinalis* L., which has a wide range of clinical applications including hemostasis, antibiosis, anti-inflammation and anti-oxidation. This study investigated the effect of ziyuglycoside II on the growth of human breast carcinoma MDA-MB-435 cells for the first time. The results showed that ziyuglycoside II could significantly inhibit the growth of MDA-MB-435 cells through blocking cell cycle progression at G0/G1 and S phase as well as via inducing cell apoptosis. Accumulation of reactive oxygen species (ROS) was observed in the progression of cell cycle arrest, which was associated with the increased expression of cell cycle regulating factors, p53 and p21. Subsequent apoptosis induced by ziyuglycoside II was accompanied with the activation of mitochondrial pathway, in particular a decreased mitochondrial membrane potential (MMP) as well as increased Bax/Bcl-2 ratio, cytochrome c release and the activity of caspase-3 and caspase-9. In conclusion, our study was the first to report that ziyuglycoside II has inhibitory effect on the growth of MDA-MB-435 cells, which might become a potential therapeutic approach of breast cancer in the future.

## 1. Introduction

Breast cancer is one of the most prevalent cancers in the world, and is also the leading cause of cancer death in women. The incidence and mortality of breast cancer is five times higher in Western countries than that of Asian countries, furthermore, it was also noticed that Asian migrants to the United States eventually progress to the same incidence of breast cancer as their host country [1]. Currently, breast cancer is mainly controlled by surgery and radiotherapy and is frequently supported by adjuvant chemo- or hormone therapies. However, this disease is highly resistant to chemotherapy and there is still no effective cure for patients with advanced stages of the disease, especially in cases of hormone-independent cancer [2]. Therefore, there are emerging needs of novel and effective regimens against breast cancer.

Chinese herbal medicine has been proved to be a valuable resource of potential anticancer agents with minimal toxicity [3,4]. Radix Sanguisorbae is the dried root of *Sanguisorba officinalis* L., which is widely distributed in the north temperate zone of Asia and Europe, particularly in China [5–7]. Radix Sanguisorbae contains various chemical compounds including tannin (17%) and triterpenoid saponins (2.4%~4.0%). Ziyuglycoside II (3β-3-α-l-arabinopyranosyloxy-19-hydroxyurs-12-*en*-28-oic acid) was the major ingredient of triterpenoid saponins exacted from Radix Sanguisorbae, which have been reported to have a wide range of clinical applications including hemostasis, antibiosis, anti-inflammation and anti-oxidation [8]. However, the anticancer effect of ziyuglycoside II on human breast carcinoma has never been reported elsewhere.

In this study, the inhibitory effect of ziyuglycoside II on the growth of MDA-MB-435 cells was reported for the first time. Such an aggressive breast carcinoma cell line has been widely used in the biological and molecular studies of breast cancer [9,10]. Furthermore, the underlying molecular mechanisms of such effects were also investigated which focused on the cell cycle distribution and apoptosis.

## 2. Results and Discussion

### 2.1. Effect of Ziyuglycoside II on Cell Growth

**Figure 1:**
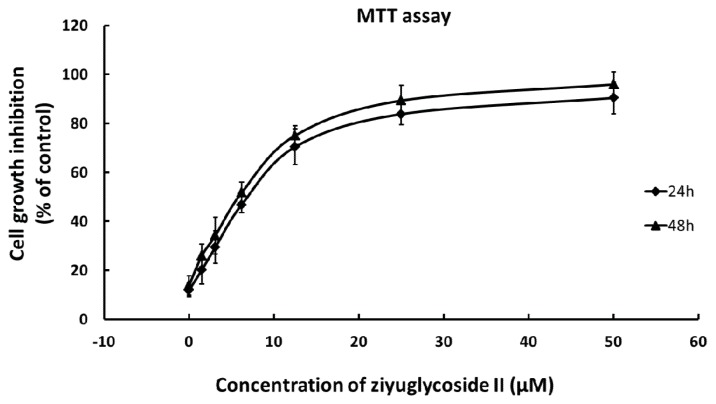
The inhibitory effect of ziyuglycoside II on the cell growth of MDA-MB-435 cells at 24 h and 48 h. All data were expressed as mean ± SD of three experiments and each experiment included triplicate repeats.

Cell growth inhibition was determined by MTT assay. As shown in Figure 1, the growth of MDA-MB-435 cells was inhibited by ziyuglycoside II in a dose-dependent manner. The IC_50_ of ziyuglycoside II at 24 h and 48 h was 5.92 μM and 4.74 μM, respectively. The effect of ziyuglycoside II on MDA-MB-435 at 48 h was similar to that of 24 h, so 24 h was chosen as the time-point for the subsequent experiments.

### 2.2. Effect of Ziyuglycoside II on Cell Cycle and Apoptosis

**Figure 2:**
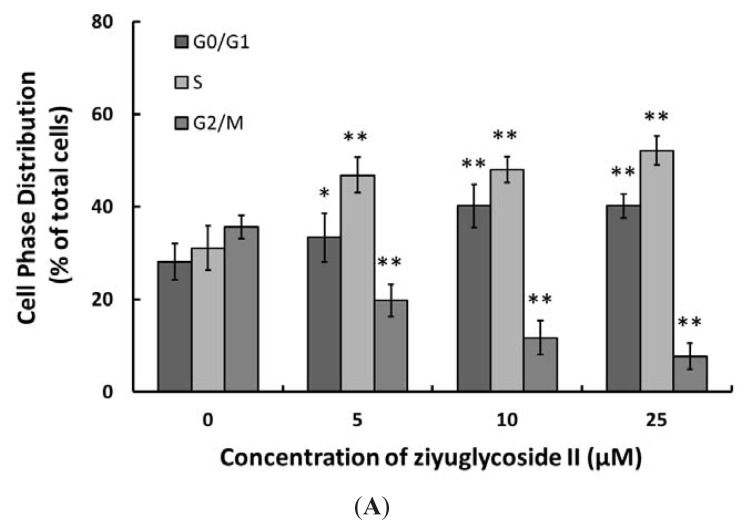

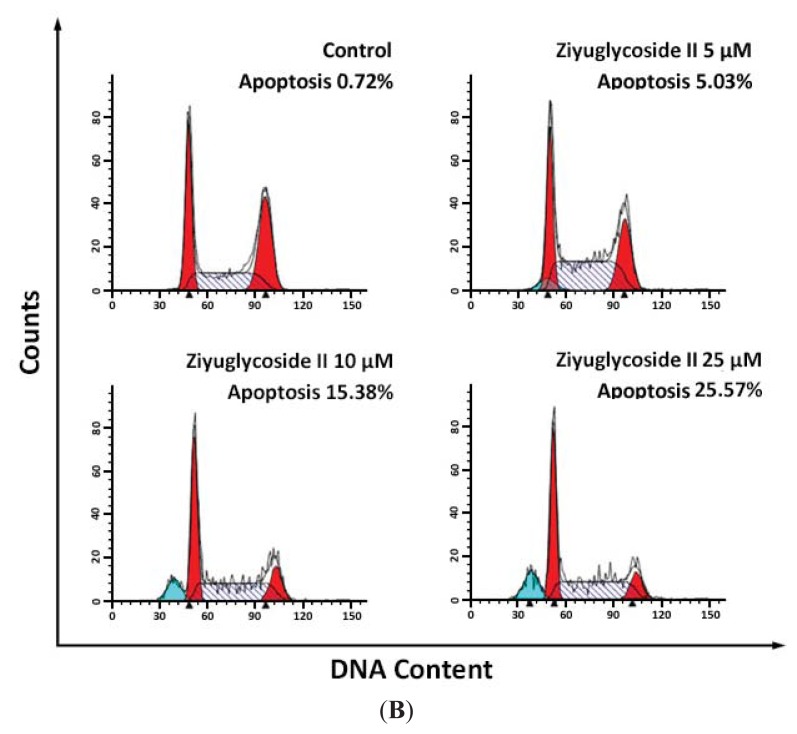
The effect of ziyuglycoside II on cell cycle distribution and apoptosis of MDA-MB-435 cells. Cells were incubated with various concentrations of ziyuglycoside II for 24 h, cell cycle distribution (**A**) and apoptosis (**B**) were assessed by flow cytometric analysis as described in Materials and Methods. All data were expressed as mean ± SD of three experiments and each experiment included triplicate repeats. * *p* < 0.05, ** *p* < 0.01 *vs.* control.

To examine the molecular mechanism of ziyuglycoside II-mediated cell growth inhibition on MDA-MB-435 cells, cell cycle distribution and apoptosis were evaluated by flow cytometric analysis. As shown in Figure 2A, ziyuglycoside II induced G0/G1 and S phase arrest at 24 h. Compared to the control group, 25 μM ziyuglycoside II increased the cell population at the G0/G1 phase from 28.12% ± 3.86% to 41.20% ± 2.61% (*p* < 0.01) and the population at the S phase from 31.08% ± 4.81% to 52.12% ± 3.13% (*p* < 0.01). We also assessed the inductive effect of ziyuglycoside II on the apoptosis of MDA-MB-435 cells. Our results indicated that the apoptotic rate was significantly increased with the treatment of ziyuglycoside II at 5 μM (5.03% ± 1.26%, *p* < 0.01), 10 μM (15.38% ± 2.87%, *p* < 0.01) and 25 μM (25.57% ± 1.73%, *p* < 0.01) for 24 h in comparison to that of the control (0.07% ± 0.05%) (Figure 2B).

### 2.3. Effect of Ziyuglycoside II on the Generation of ROS

**Figure 3:**
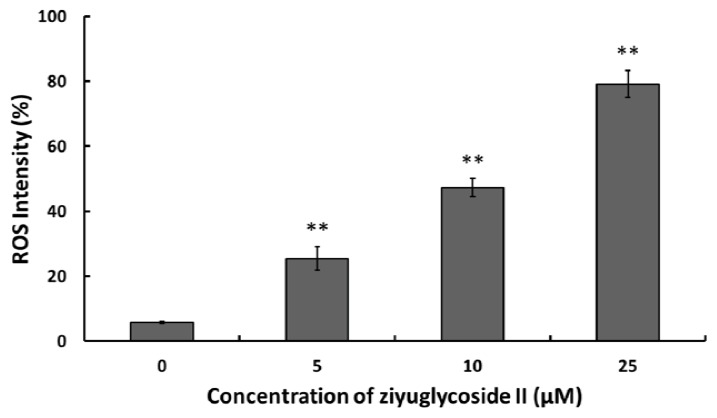
The effect of ziyuglycoside II on the generation of ROS in MDA-MB-435 cells. Cells were incubated with various concentrations of ziyuglycoside II for 6 h and then harvested for ROS determination as described in Materials and Methods. All data were expressed as mean ± SD of three experiments and each experiment included triplicate repeats. * *p* < 0.05, ** *p* < 0.01 *vs.* control.

ROS from mitochondria and other cellular sources have traditionally been considered as toxic by-products of metabolism [11], meanwhile, ROS has been reported to have apoptotic induction effects [12]. In this study, the ROS level was significantly elevated in the ziyuglycoside II-treated groups when compared to that of the control. Ziyuglycoside II induced the accumulation of ROS in MDA-MB-435 cells in a dose dependent manner (Figure 3).

### 2.4. Effect of Ziyuglycoside II on Expressions of p53 and p21

**Figure 4:**
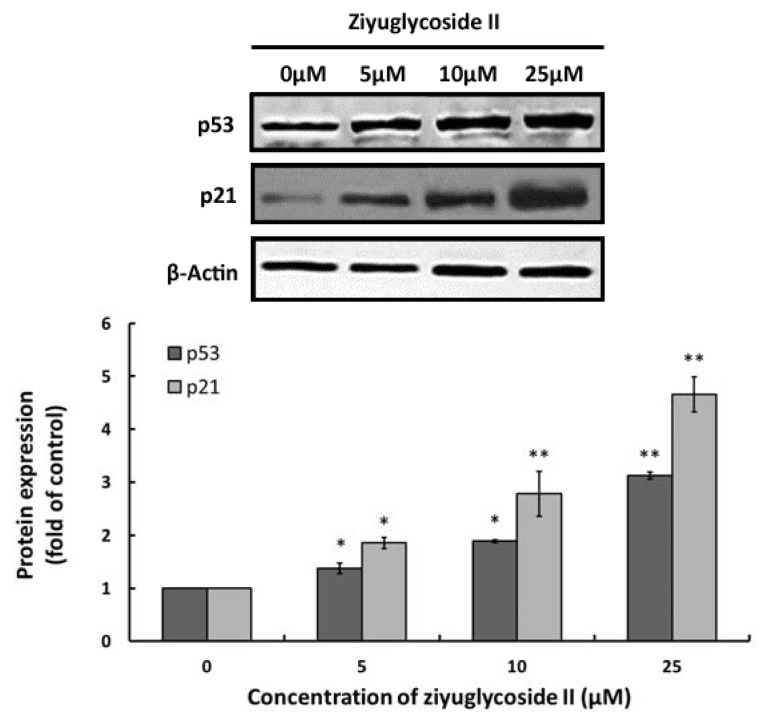
The effect of ziyuglycoside II on expressions of p53 and p21. Cells were incubated with various concentrations of ziyuglycoside II for 24 h and the expressions of p53 and p21 were assessed by Western blot analysis. (**Upper panel**) Immunobloting of p53 and p21, while β-actin was probed as the protein loading control; (**Lower panel**): Densitometry analysis of p53 and p21 protein expression. All data were expressed as mean ± SD of three experiments and each experiment included triplicate repeats. * *p* < 0.05, ** *p* < 0.01 *vs.* control.

To determine the involvement of cell cycle regulating factors in the ziyuglycoside II-induced cell cycle arrest, we evaluated the protein expressions of two key protein factors, p53 and p21, through western blot analysis. Our results revealed that the treatment of ziyuglycoside II on MDA-MB-435 cells resulted in increased expressions of both p53 and p21, which effect was dose-dependent (Figure 4).

### 2.5. Effect of Ziyuglycoside II on the MMP

**Figure 5:**
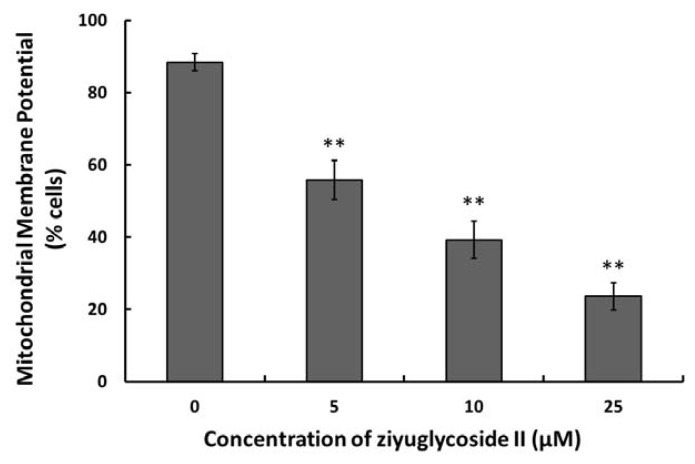
The effect of ziyuglycoside II on the mitochondria membrane potential (MMP) in MDA-MB-435 cells. Cells were incubated with various concentrations of ziyuglycoside II for 24 h and then harvested for MMP determination as described in Materials and Methods. All data were expressed as mean ± SD of three experiments and each experiment included triplicate repeats. * *p* < 0.05, ** *p* < 0.01 *vs.* control.

Loss of mitochondria membrane potential (MMP) has been reported as an early event in some apoptotic processes [13]. Our data demonstrated that ziyuglycoside II treatment led to a decreased MMP in the MDA-MB-435 cells in a dose dependent manner (Figure 5).

### 2.6. Effect of Ziyuglycoside II on the Expressions of Bax, Bcl-2 and Cytochrome C

**Figure 6:**
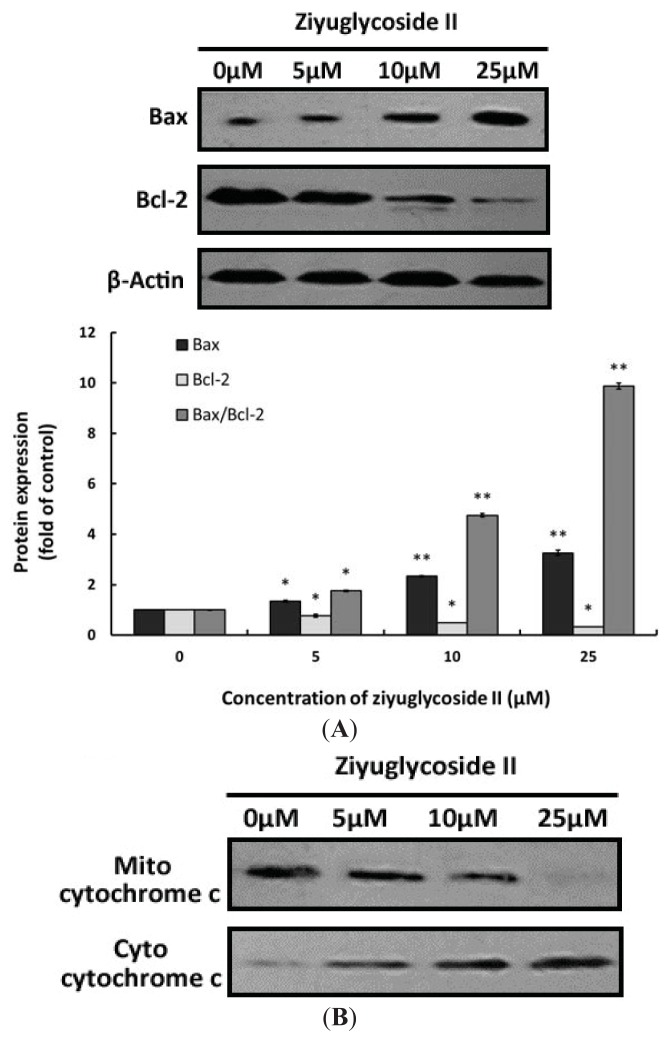
The effect of ziyuglycoside II on expressions of apoptosis related proteins. Cells were incubated with various concentrations of ziyuglycoside II for 24 h and then the expressions of Bax, Bcl-2 and cytochrome c were assessed by western blot analysis. (**A**) Protein expression of Bax and Bcl-2: **Upper panel**: immunobloting of Bax and Bcl-2, while β-actin was probed as the protein loading control; **Lower panel**: densitometry analysis of Bax, Bcl-2 and Bax/Bcl-2 ratio. All data were expressed as mean ± SD of three experiments and each experiment included triplicate repeats. * *p* < 0.05, ** *p* < 0.01 *vs.* control; (**B**) Immunobloting of mito-cytochrome c and cyto-cytochrome c. All data were representative of three independent experiments.

We also investigated the expressions of apoptosis-related proteins, particularly Bax and Bcl-2, in MDA-MB-435 cells under the treatment of ziyuglycoside II for 24 h. Figure 6A showed that ziyuglycoside II treatment could up-regulate the expression of Bax but down-regulate the expression of Bcl-2 in a dose-dependent manner. The ratio of Bax/Bcl-2 increased approximate 9.88 fold compared to the control.

Cytochrome c release from mitochondrion to cytosol has been predicted as one of the major envisages of cell apoptosis [14,15], we also examined the protein expressions of mito-cytochrome c and cyto-cytochrome c. Our results indicated that the treatment of ziyuglycoside II might have also induced the release of cytochrome c from mitochondrion into cytosol in a dose-dependent manner (Figure 6B).

### 2.7. Effect of Ziyuglycoside II on the Activity of Caspase-3 and Caspase-9

**Figure 7:**
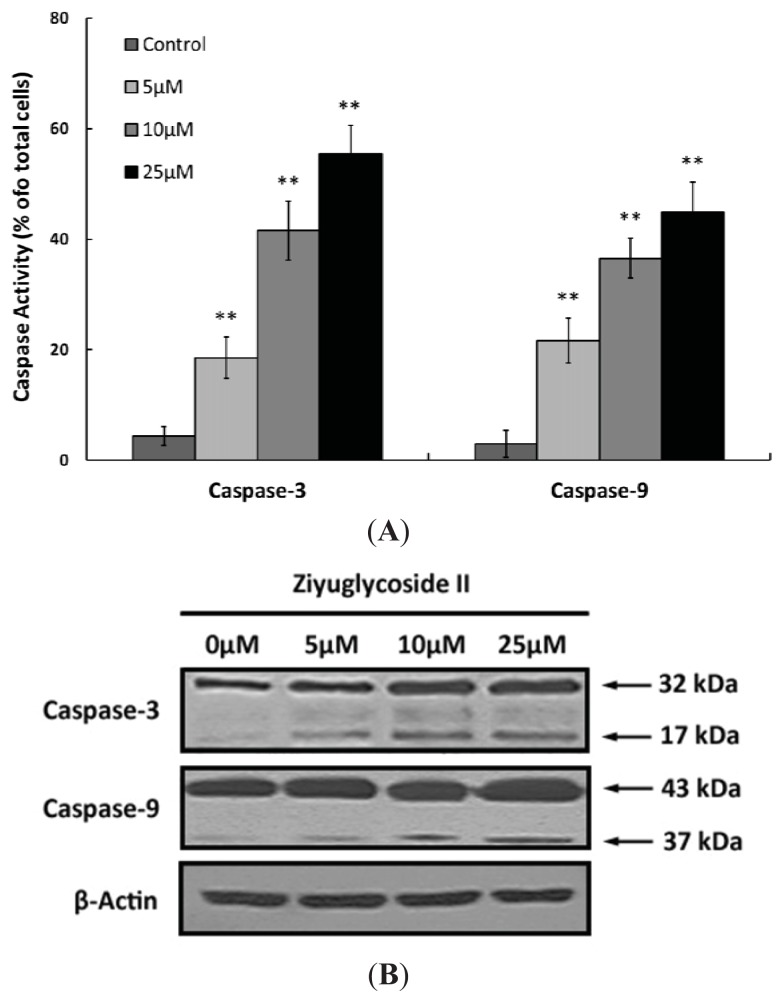
The effect of ziyuglycoside II on the activity and protein cleavage of caspase-3 and caspase-9. Cells were incubated with various concentrations of ziyuglycoside II for 24 h and then further analyzed for the activity and protein cleavage of caspase-3 and caspase-9. (**A**) The activity of caspase-3 and caspase-9 were determined as described in Materials and Methods. All data were expressed as mean ± SD of three experiments and each experiment included triplicate repeats. * *p* < 0.05, ** *p* < 0.01 *vs.* control; (**B**) The cleavage of caspase-3 and caspase-9 were assessed by western blot analysis. All data were representative of three independent experiments.

The release of cytochrome c could activate caspase-9, a cysteine protease. Caspase-9 can then go on to activate caspase-3, which is responsible for destroying the cells [16]. To assess whether the caspase pathway is involved in the ziyuglycoside II-induced apoptosis in MDA-MB-435 cells, the activities of caspase-3 and caspase-9 were assessed accordingly. As shown in Figure 7A, ziyuglycoside II treatment could activate the function of caspase-3 and caspase-9. Furthermore, Figure 7B showed that ziyuglycoside II treatment also resulted in the pronounced increase of caspase-3 and caspase-9 cleavage in MDA-MB-435 cells. Both effects mentioned above were in a dose dependent manner.

### 2.8. Discussion

*Sanguisorba officinalis* L. has been effectively used for the treatment of inflammation and tumor [5–7]. Ziyuglycoside II is one of the major active compounds of *Sanguisorba officinalis* L., the anti-cancer activity of ziyuglycoside II has never been reported before. Our study was the first to investigate the molecular effects of ziyuglycoside II on human breast carcinoma MDA-MB-435 cells. The results demonstrated that ziyuglycoside II could induce a significant dose-dependent inhibition of MDA-MB-435 cell growth (Figure 1). The IC_50_ of ziyuglycoside II was 5.92 μM at 24 h. Further analysis revealed ziyuglycoside II inhibited the growth of MDA-MB-435 cells mainly through blocking cell cycle progression at G0/G1 and S phase as well as via inducing cell apoptosis.

Furthermore, we explored the molecular mechanisms underlying the ziyuglycoside II induced cell cycle arresting in MDA-MB-435 cells. Previous studies showed that chemo preventive agents were capable of inducing cell cycle blocking and apoptosis, in part through the accumulation of ROS and the disruption of redox homeostasis [17,18]. It was also known that ROS accumulation could lead to ER stress and DNA damage that resulting in protein expressional change of the downstream proteins such as p53. p53 plays a key role in mediating cell response to various stresses, mainly through inducing or suppressing a number of genes involved in cell cycle arrest and apoptosis [19]. Modulation of cell cycle-related genes by p53 activation may mediate cell cycle arrest at one of two major cell-cycle checkpoints, in G1 near the border of S-phase (primarily determined by p21WAF1/CIP1) or in G2 before mitosis (mainly controlled by GADD45 and 14-3-3σ) [20]. P21 is a very important checkpoint gene in the cell cycle, which is also regulated by the transcription of p53; it can repair damaged cells by stopping DNA synthesis and inactivate the nuclear antigen in proliferating cells [20]. Our data showed that ziyuglycoside II induced ROS accumulation in a dose-dependent manner (Figure 3) and also up-regulated p53 and p21 expression (Figure 4). These data suggested that the cell cycle arrest induced by ziyuglycoside II might be mediated through the regulation of the cell cycle regulating factors, in particular p53 and p21.

**Figure 8:**
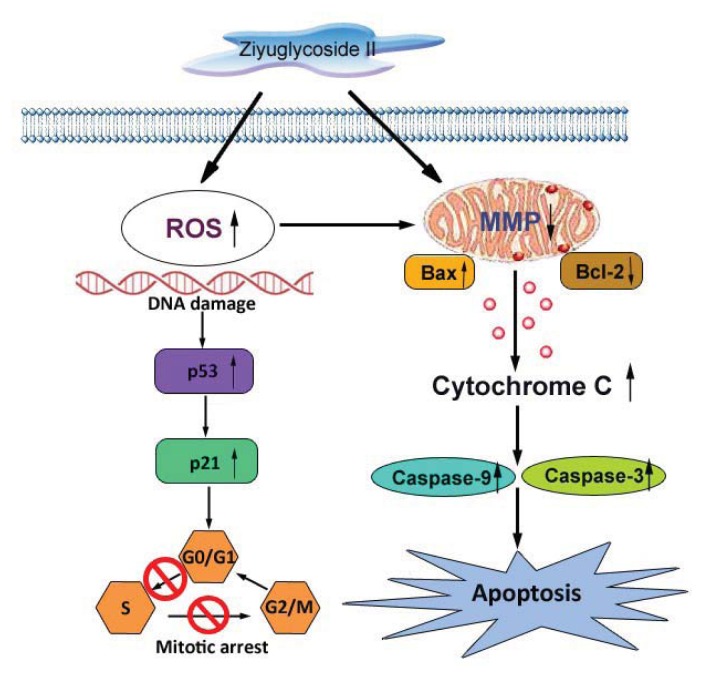
The proposed mechanisms for ziyuglycoside II induced cell cycle arrest and apoptosis in MDA-MB-435 cells. Ziyuglycoside II increased the accumulation of ROS, which resulted in the up-regulated expression of p53, p21 and Bax but down regulated expression of Bcl-2 in MDA-MB-435 cells. Consequently, the mitochondrial membrane potential (MMP) was reduced and then accelerated the release of cytochrome c into cytoplasm leading to the apoptosis of MDA-MB-435 cells via the caspase-3 and caspase-9 dependent pathway.

The essential cancer therapeutic stratagem is to induce apoptosis in cancer cells, which could be divided into the extrinsic or the mitochondria-dependent pathways [16,21,22]. In our ongoing efforts to determine the apoptotic mechanism of ziyuglycoside II on MDA-MB-435 cells, we focused on the mitochondrial pathway. Unbalanced formation of ROS and antioxidants often leads to cell apoptosis and death [23]. An increased mitochondrial accumulation of ROS triggers the intrinsic pathway by increasing the permeability of the outer mitochondrial membrane, which accelerates cytochrome c moving from the intermembrane space into cytosol [24] Apoptosis is often associated with impaired mitochondrial adenine nucleotide exchange; the alternative channels in the mitochondrial outer membrane permit the transit of adenine nucleotides [25]. This process can be regulated by the expression of Bcl-2 or Bcl-xL [26,27]. The perturbation of nucleotide exchange has been postulated to lead to permeability transition and cytochrome c release. Our data demonstrated that ziyuglycoside II reduced the level of MMP (Figure 5) and increased the expression of Bax but decreased Bcl-2, thus the calculated Bax/Bcl-2 ratio was markedly higher in the ziyuglycoside II-treatment group compared to the control group (Figure 6), which then accelerated the movement of cytochrome c into cytosol. It is known that cytochrome c interacts with procaspase-9 to activate initiator caspase-9, and then activates effecter caspase-3, which is achieved by caspase-9 cleaves at specific internal Asp residues that separate the large and small subunits. In our study, the elevated cleavage of caspase-9 and caspase-3 were significantly increased in MDA-MB-435 cells treated with ziyuglycoside II in a dose dependent manner (Figure 7). The complex mechanism of ziyuglycoside II-induced cell cycle arrest and apoptosis in MDA-MB-435 cells is depicted in Figure 8. Now in clinical trials are a series of targeted agents that directly inhibit cell growth and induce cell cycle arrest and apoptosis [28–30]. An understanding of mechanisms of the cell growth inhibition is critical to understand how best to clinically develop anticancer agents, both as single agents [31] and in combination with chemotherapy [32,33]. As such, investigation of mechanisms of ziyuglycoside II induced cell cycle arrest and apoptosis in MDA-MB-435 cells may provide valuable information for its application in breast cancer therapy.

## 3. Experimental Section

### 3.1. Materials

Ziyuglycoside II was obtained from National Institute for the Control of Pharmaceutical and Biological Products (Beijing, China). Fetal bovine serum (FBS) and RPMI medium 1640 were obtained from Gibco (Grand Island, NY, USA). 3-(4,5-dimethylthiazol-2-yl)-2, 5-diphenyl tetrazolium bromide (MTT), 2,7-dichlorodihydrofluorescein diacetate (DCFH-DA) and propidium iodide (PI) were obtained from Sigma (St. Louis, MO, USA). Dimethyl sulfoxide (DMSO), sodium bicarbonate, penicillin-streptomycin, trypsin, enhanced chemiluminescence (ECL) assay kit and PVDF membrane were obtained from Beyotime (Nantong, China). Caspase Colorimetric Assay Kit was obtained from R&D Systems (Minneapolis, MN, USA). The monoclonal antibodies used in this study were listed as below: p53, Bcl-2, cytochrome c, caspase-9, caspase-3 (Oncogene Science, Cambridge MA, USA), p21 (Santa Cruz, Santa Cruz, CA, USA), Bax (Biomol, Farmingdale, PA, USA) and HRP conjugated goat anti rabbit secondary antibody (Santa Cruz, Santa Cruz, CA, USA).

### 3.2. Cell Culture

Human MDA-MB-435 breast carcinoma cells were obtained from the American Tissue Culture Collection. MDA-MB-435 cells were cultured in RPMI 1640 medium supplemented with 10% fetal bovine serum and 1% penicillin-streptomycin at 37 °C in a humidified atmosphere containing 5% CO_2_.

### 3.3. Cell Growth Assay

To assess the effect of ziyuglycoside II on cell growth, cells (1 × 10^4^ cells/well) were seeded in 96-well plates with RPMI 1640 containing 10% fetal bovine serum. After incubated for 24 h, cells were treated with various concentrations of ziyuglycoside II (0, 1.5, 3.1, 6.2, 12.5, 25, 50 μM) for 24 h or 48 h. At the end of time points, cell proliferation was measured by MTT assay as mentioned before [34]. Cell inhibition was expressed as a percentage of the control. The experiment was done in triplicate repeats and the results are indicative of three independent studies.

### 3.4. Cell Cycle Distribution and Apoptosis Analysis

Cells (1 × 10^5^ cells/well) were seeded in 6-well plates with 10% fetal bovine serum incubated overnight, and then serum starved for 24 h to synchronize them in the G0 phase of cell cycle. Synchronous populations of cells were incubated with various concentrations of ziyuglycoside II (0, 5, 10 and 25 μM) for 24 h, washed twice with ice-cold Phosphate Buffered Saline (PBS) (pH 7.4) and then centrifuged. The pellet was fixed in 75% (*v*/*v*) ethanol for 1 h at 4 °C, washed once with ice-cold PBS, and then suspended in cold PBS supplied with propidium iodide (PI) solution (50 μg/mL) and Ribonuclease A (RNase A) (0.1 mg/mL) for 30 min in the dark. Cell cycle and apoptosis were assessed using a flow cytometer (Becton, Dickinson and Company, Franklin Lakes, NJ, USA).

### 3.5. Intracellular Reactive Oxygen Species (ROS) Detection

The level of ROS in MDA-MB-435 cells was examined by flow cytometry, using DCFH-DA staining. Cells were treated with various concentrations of ziyuglycoside II (0, 5, 10 and 25 μM) for 6 h at 37 °C in a humidified atmosphere containing 5% CO_2_. Then cells were harvested, washed and re-suspended in 500 μL of DCFH-DA (10 μM), and then incubated at 37 °C for 30 min before analyzed by flow cytometry [35].

### 3.6. Measurement of Mitochondrial Membrane Potential (MMP)

The level of cell MMP in MDA-MB-435 cells was determined by flow cytometry, using DiOC6 (4 μM) staining. Cells were treated with various concentrations of ziyuglycoside II (0, 5, 10 and 25 μM) for 24 h, harvested, washed, re-suspended in 500 μL of DiOC6 (4 μM) and incubated at 37 °C for 30 min. The level of MMP was then analyzed by flow cytometry [36].

### 3.7. Measurement of Cytochrome C Release

To determine whether ziyuglycoside II induces the release of cytochrome c from mitochondria, cells were treated with various concentrations of ziyuglycoside II (0, 5, 10 and 25 μM) for 24 h. Using Apo Alert Cell Fractionation Kit (Clontech, Laboratories Inc., Mountain View, CA, USA), mitochondrial and cytosolic fractions were extracted from the treated and untreated cells. The level of cytochrome c was determined using a monoclonal antibody of cytochrome c through western blot as described below.

### 3.8. Western Blotting Analysis

Cells were incubated with various concentrations of ziyuglycoside II (0, 5, 10 and 25 μM) for 24 h. Approximately 1 × 10^6^ cells were collected and lysed in ice-cold RIPA buffer (50 mM Tris-HCl, 150 mM NaCl, 1 mM ethylene glycol tetraacetic acid (EGTA), 1 mM ethylene diamine tetraacetic acid (EDTA), 20 mM NaF, 100 mM Na_3_VO_4_, 1% Nonidet P-40 (NP-40), 1% Triton X-100, 1 mM phenylmethylsulfonyl fluoride (PMSF), 10 mg/mL Aprotinin and 10 mg/mL Leupeptin) for 30 min. Protein concentration was determined by the Bradford method [37]. Cell lysates were electrophoresed on a 10% SDS polyacrylamide gel and transferred onto polyvinylidenefluoride (PVDF) membrane. After blocking with 5% bovine serum albumin (BSA) in the mixture of Tris-Buffered Saline and Tween-20 (TBST) for 1 h, membranes were incubated with the primary antibodies overnight and followed by incubation with the secondary antibody for 1 h at room temperature. Protein bands were visualized using the ECL assay kit. The density of each band was normalized as to the expression of β-actin.

### 3.9. Caspase-3 and -9 Activity Assays

Approximately 2 × 10^6^ MDA-MB-435 cells were plated onto 6-well plates and treated with various concentrations of ziyuglycoside II (0, 5, 10 and 25 μM) for 24 h. Caspase-3 and caspase-9 activity was assessed according to the manufacturer’s instruction. Cells were collected and lysed in caspase assay buffer containing 50 mM Tris-HCl (pH 7.4), 1 mM EDTA, 10 mM EGTA, 10 mM digitonin and 2 mM Dithiothreitol (DTT). Then the ice-cold cell mixtures were centrifuged and the suspensions were transferred into new tubes. After quantified, 5 μg proteins were incubated with caspase-3- and caspase-9 specific substrates (Ac-DEVD-pNA for caspase-3 and Ac-LEHD-pNA for caspase-9; R&D Systems, Minneapolis, MN, USA) for 1 h at 37 °C, respectively. The caspase activity was determined by measuring OD_405_ of the released pNA [38].

### 3.10. Statistical Analysis

Biostatistical analyses were done using the SPSS 16.0 software package (Statistical Product and Services Solutions, Chicago, IL, USA; Available online: http://download.pinggu.org/spss/SPSSv16.0.rar). All experiments were repeated three times. Results of multiple experiments were expressed as mean ± SD. A *p* value less than 0.05 was accepted as statistically significant.

## 4. Conclusions

In summary, our study demonstrated that ziyuglycoside II inhibited the growth of breast carcinoma MDA-MB-435 cells through the induction of cell cycle arrest and apoptosis in the mitochondria-dependent pathway (Figure 8). Ziyuglycoside II increased the accumulation of ROS, which resulted in the up-regulated expression of p53, p21 and Bax but down regulated expression of Bcl-2 in MDA-MB-435 cells. Consequently, the mitochondrial membrane potential (MMP) was reduced and then accelerated the release of cytochrome c into cytoplasm leading to the apoptosis of MDA-MB-435 cells via the caspase-3 and caspase-9 dependent pathway. Therefore, ziyuglycoside II may become a promising anti-cancer agent to treat breast cancer upon the therapeutic confirmation in *in vivo* models.
